# Modelling the surface free energy parameters of polyurethane coats—part 2. Waterborne coats obtained from cationomer polyurethanes

**DOI:** 10.1007/s00396-013-3156-x

**Published:** 2014-01-14

**Authors:** Piotr Król, Bożena Król, Jaromir B. Lechowicz

**Affiliations:** Department of Polymer Science, Faculty of Chemistry, Rzeszów University of Technology, Al. Powstańców Warszawy 6, 35-959 Rzeszów, Poland

**Keywords:** Waterborne cationic polyurethane, Surface free energy parameters, Additive model for the surface free energy

## Abstract

Polyurethane cationomer coats were synthesised on the basis of typical diisocyanates, properly selected polyether polyols, HO-tertiary amines and HCOOH as quaternisation reagents. The values of their surface free energy (SFE) parameters were obtained by the van Oss-Good method, with the use of the contact angle values which had been found by the goniometric method. Based on the obtained findings, empirical models were developed which made it possible to anticipate the effects of the raw material types on the SFE values of the produced coats. The possibility was noted to adjust the SFE values within 25–50 mJ/m^2^ by selecting carefully suitable parent substances. The principal consequences for the formation of improved hydrophobicity coats, applicable inter alia specialised protective coatings, were found to come not only from diisocyanate and polyol types but also from the alkylammonium cation structure which results from the use of different tertiary amines. The fundamental SFE lowering effect was noted when tertiary amines with 0–15 % of the 2,2,3,3-tetrafluoro-1,4-butanediol as a fluorinated chain extender was incorporated into polymer chains.

## Introduction

The coatings obtained from cationomer polyurethanes (more frequently termed “cationic polyurethanes” in reference papers) were found applicable as waterborne polyurethanes in the past years for the production of environmentally friendly lacquers, adhesives and protective coatings, coating base paper, ink jet printing base paper, etc. [1–3]. The definite majority of the cationic polyurethanes referred herein which is specific is obtained by reacting typical diisocyanates, polyols and *N*-methyldiethanoloamine (*N*-MDA); they are then subjected to further quaternisation in an aqueous medium with the use of an organic acid in most cases [1–6]. The number of ionic groups present within a polymer is critical when one wants to achieve appropriate mechanical properties, surface properties, thermal stability or biodegradability of the coats obtained from polyurethane cationomers. That number is decisive for the ionic strength of the aqueous dispersion of an ionomer which contains bulky alkylammonium cations in its molecules [1–3, 7]. Because of that, polyurethane cationomers may also be used as components in stable aqueous dispersions for free radical polymerisation of styrene and acrylic monomers which yields poly(urethane-styrene-acrylic) copolymers with *core*-*shell* structures. They turned out applicable as water-dilutable emulsion paints and protective coatings [8, 9]. Research was also conducted on the use of PU cationomers in the synthesis of polymeric nanocomposites incorporating intercalated or exfoliated layer silicate clays and composites which involve graphenes [10, 11]. When formulating hydrophobic coats, it is advantageous to incorporate fluorine atoms into polyurethane chains, including polyurethane ionomers that can be done in a few ways. Fluorine may be added with the isocyanate reactant, which is relatively difficult. Also, it may be added as a fluorinated derivative of polyol or as a chain extender in the form of generally available 2,2,3,3-tetrafluoro-1,4-butanediol (TFBD) [5, 12–15].

Not only *N*-MDA is used for the manufacture of alkylammonium cations but also other more water-repellent tertiary amines or amino acids like, for example, lysine may also be used [16, 17]. Cationic biodegradable multiblock poly(ε-caprolactone urethane)s have been synthesised which contain gemini quaternary ammonium side groups on the hard segments. To obtain these polyurethanes, a new l-lysine-derivatised diamine containing gemini quaternary ammonium side groups was first synthesised [17].

Radicals derived, e.g. from alkyl haloids, which are added when the final aqueous dispersion is produced, may additionally reduce polarity of the coats. New counterions are obtained in that way which are more active in many cases than anions derived from organic acids or from frequently employed hydrochloric acid [18].

Having in mind structural variety of cationomers and their growing importance in the production of environmentally friendly polyurethane dispersions, we started the research programme which was intended to develop empirical models to describe the structural effects of cationomers on surface free energy (SFE) values of polyurethane coats formed from those cationomers. SFE makes a measure which represents well the chemical nature of each polymer coats or film from the viewpoint of its water-repellent performance. Earlier, we conducted similar studies for the coats which had been obtained from polyurethane elastomers applied as solutions in organic solvents [19]. The problem turned out much more difficult in the present case because of higher structural complexity of cationomer chains. Moreover, the models had to consider not only polyurethane structural fragments which were derived from diisocyanates, polyols and chain extenders but also those derived from tertiary amines and counterions as they made considerable contributions to the structures of alkylammonium cations. Similar to the approach presented in [19], sets of raw materials which were principally decisive for the chemical structures of polyurethane cationomer chains were assumed as independent variables for a given category.

Since the reaction system was more complex, we unfortunately could not develop linear models, as was the case for polyurethanes. Structural parameters *κ* ^exp^ were independent variables in the previous models; those parameters could be found with the use of the ^1^H NMR spectral analysis [19]. All the same, we verified chemical structures of all polyurethane cationomers covered by the present study with the use of spectral methods (FT IR, ^1^H NMR and ^13^C NMR) and ^19^F NMR spectroscopy if needed [15, 20]. This study also covered a number of cationomer coats which had been synthesised earlier and for which chemical structures and SFE parameters had been determined precisely. The references to those publications were given in Table 1. Some cationomers, however, had to be synthesised additionally for the needs of this study, and a few syntheses were conducted once more and a few SFE measurements were taken once more to verify our earlier findings.

**Table 1:** Chemical compositions of synthesised polyurethane cationomers

Sample no.	Type of diisocy-anate	Type of polyol	Type of tertiary amine with wt % of TFBD $\left({\overline{\mathrm{M}}}_{\mathrm{calc}}\right)$	*N*_quaternary_^+^ content wt %	Fluorine content, wt %	Reference
1 and 1a^a^	MDI	POG 600	NMDA + 5 wt % TFBD (120.18)	2.15	0.54	[15]
2 and 2a	MDI	POG 600	NMDA + 10 % TFBD (122.05)	2.27	1.09	[15]
3	MDI	POG 600	NMDA + 15 % TFBD (124.09)	2. 35	1.66	[15]
4	MDI	POG 600	NBDA	2.39	0	[15]
5	MDI	POG 600	NBDA + 5 % TFBD (161.28)	2.49	0.68	[15]
6 and 6a	MDI	POG 600	NBDA + 10 % TFBD (161.33)	2.23	1.36	[15]
7	MDI	POG 600	NBDA + 15 wt % TFBD (161.42)	2.12	2.04	[15]
8 and 8a	MDI	PPG 450	NMDA	1.19	0	[7, 16]
9	MDI	PPG 450	NBDA	1.26	0	[16]
10	IPDI	POG 600	NMDA	1,99	0	[16]
11	IPDI	POG 600	NMDA + 5 % TFBD (120.18)	2.10	0.68	[15]
12	IPDI	POG 600	NMDA + 10 % TFBD (122.05)	2.46	1.09	[24]
13	IPDI	POG 600	NMDA + 15 % TFBD (124.09)	2.56	1.79	[24]
14	IPDI	POG 600	NBDA + 15 % TFBD (161.42)	2.25	2.15	
15 and 15a	IPDI	POG 600	NBDA	2.55	0	
16	IPDI	POG 600	NBDA + 5 % TFBD (161.28)	2.50	0.71	
17	IPDI	POG 600	NBDA + 10 % TFBD (161.33)	2.38	1.43	
18	IPDI	PPG 450	NMDA	2.94	0	

^a^Syntheses of the sample nos. 1, 2, 6, 8 and 15 are repeated and new samples were marked with a letter “a”

## Experimental

### Reagents

The following reagents used for the synthesis of polyurethane cationomers were characterised in part I of our work [19]:

- 4,4′-Methylenebis(phenyl isocyanate) (*M* = 250.25) (MDI)
- Isophorone diisocyanate, [5-isocyanato-1-(isocyanatomethyl)-1,3,3-trimethylcyclohexane] (*M* = 222.28) (IPDI)
- Polyoxyethylene glycols, *M* = 600
- 2,2,3,3-Tetrafluoro-1,4-butanediol (*M* = 162.08) (TFBD)
- 1,6-Hexamethylenediamine (HMDA) (*M* = 116.21)
- *N*-methyldiethanolamine (*M* = 119.16) (NMDA)
- *N*-butyldiethanolamine (*M* = 161.24) (NBDA)
- Dibutyl tin dilaurate (DBTDL)

It is also regarding analytical reagents:

- Dibutylamine, diiodomethane, formamide and redistilled water
- Formic acid (HCOOH), 99 %, analytically pure (*M* = 46.03), POCh S.A., Gliwice, Poland

### Method for the synthesis of polyurethane cationomer coatings

The cationomers used in our research were synthesised in a four-staged polyaddition process: At stage 1, urethane-isocyanate prepolymer was synthesised in the reaction of diisocyanate (B) and polyether (A):

1 $$ \mathrm{A}+2\mathrm{B}\to \mathrm{BAB} $$

At stage 2, the prepolymer was reacted with *N*-alkyldiethanolamine (X):

2 $$ \mathrm{nBAB}+\mathrm{nX}\to -{\left(\mathrm{XBABXB}\right)}_{\mathrm{n}}- $$

Alkylammonium cations were produced at stage 3 by reaction of tertiary amino groups with HCOOH.

At stage 4, redistilled water with a small amount of HMDA was added under intensive agitation conditions. That stage was intended not only to produce the water dispersion (30–40 wt ) but also cationomer chains with the residual –NCO groups were subjected to extension at the same time in the reaction between –NCO groups and water:3or in the reaction between residual –NCO groups and HMDA:4


Fluorine was added to the molecular chains of the same cationomers (Table 1) in the synthesis of prepolymers in the reaction of the isocyanate prepolymer BAB and the mixture of *N*-MDA or *N*-BDA with 0, 5, 10 or 15 wt% of TFBD, respectively. The fluorine contents in those cationomers, as calculated from stoichiometry, were from 0.54 up to 2.04 %.

The detailed description of the cationomer synthesis processes was provided in [14]. The polymer coats for further tests were formed by applying the abovementioned dispersions to a nonpolar surface of poly(tetrafluoroethylene) (PTFE) and evaporation of water by air-drying at 20 °C. The coats were then subjected to seasoning under such conditions over 10 days. Chemical compositions of cationomers can be found in Table 1. The final product obtained after prepolymer extension was a cationomer linear polyurethane, as expected. Its chains were composed of structural units which formed soft polyol segments A and hard urethane and urea segments and ionic groups formed of alkylammonium cations and HCOO^–^ counterions. The ionic strength of the obtained cationomers may be inferred from the number of integrated N^+^ quaternary cations; that number—as calculated from stoichiometry—was from 1.19 up to 2.94 wt% (Table 1). That load was counterbalanced by the equimolar number of counteranions. Thus, strong polar, ionic and acid-base interactions could be expected. Also, numerous hydrogen bonds could be formed between soft and rigid segments which were present in the polymer chains. Those interactions were reflected—as it was demonstrated afterwards—in relatively high-surface free energy values for the produced cationomer films. The SFE values for F-containing coats turned out much lower, as was expected.

### Surface free energy

Physical parameters of surface free energy of a solid (*γ*_*S*_) were found in the present study on the basis of the van Oss-Good method [21]. This method is much more proper in many cases than the Neumann method although its procedure requires the use of three measuring liquids for evaluation of SFE of polymer materials. Admittedly, the latter method is based on contact angle measurements with the use of only one measuring liquid, but its theoretical justification is still an arousing controversy [22]. The van Oss-Good model assumes that the surface free energy *γ*_S,L_ may be presented as a sum of two components:

5 $$ {\gamma}_{S,L}={\gamma}_{S,L}^{LW}+{\gamma}_{S,L}^{AB} $$

Where

*γ*_*S*_^*LW*^: surface energy connected with long-range interactions (dispersion, polar and induction interactions)
*γ*_*S*_^*AB*^: surface energy connected with acid-base interactions, as results from the Lewis theory.

Equation (5) is generally applicable both to a solid, and the marking with the subscript *S* is used then, and to a wetting liquid (standard liquid or tested liquid) in which case the subscript *L* is employed.

Let us use the symbol *γ*_S_^+^ for the component of *γ*_*S*_^*AB*^ which is responsible for the surface free energy of the Lewis acid and the symbol *γ*_*S*_^−^ for the component representing the Lewis base. On the basis of the Berthelot theory, which assumes that interactions between molecules of different bodies located on a surface are equal to the geometric mean of interactions between molecules within each of those bodies, one can formulate the following relations:

- For bipolar substances (liquids and surfaces of solids), which can be equivalent to synthesised PU ionomers—present in the form of aqueous dispersions or coatings:
  6 $$ {\gamma}_i^{AB}=2\sqrt{\gamma_i^{+}\cdot {\gamma}_i^{-}} $$
- For nonpolar liquids and surfaces of solids (diiodomethane or PTFE):
  7 $$ {\gamma}_i^{AB}=0 $$

Where *i* = *S*, solid or *L*, liquid.

The SFE parameters for solids (*S*) and for liquids (*L*) interacting with those solids should satisfy the van Oss-Good equation:

8 $$ \sqrt{\gamma_S^{LW}\cdot {\gamma}_L^{LW}}+\sqrt{\gamma_S^{+}\cdot {\gamma}_L^{-}}+\sqrt{\gamma_S^{-}\cdot {\gamma}_L^{+}}=\frac{1}{2}{\gamma}_L\cdot \left(1+ \cos \varTheta \right) $$

Where *Θ* is the experimentally found wetting angle between a liquid drop and a solid surface under investigation. So, wetting angles *Θ* were first measured for the surfaces of cationomer coatings with the use of three model liquids (redistilled water, diiodomethane and formamide) with known parameters of γ_*L*_, *γ*_*L*_^*LW*^, *γ*_*L*_^+^ and *γ*_*L*_^−^ (Table 2), and then Eq. (8) was used to calculate the values of *γ*_*S*_^*LW*^, *γ*_*S*_^+^ and *γ*_*S*_^−^ for the studied cationomers. The values of γ_*S*_^*AB*^ were calculated from the Eq. (5), while the values of *γ*_*S*_^*exp*^ from Eq. (5). Only *γ*_*S*_^exp^ values were utilised in numerical computations.

**Table 2:** Surface properties of the model measuring fluids [22]

Model measuring liquid	Surface free energy parameters (0.001 J/m^2^)				
	γ_L_	*γ*_L_^*LW*^	*γ*_L_^*AB*^	*γ*_L_^−^	*γ*_L_^+^
Redistilled water	72.8	21.8	51	25.5	25.5
Formamide	58.0	39.0	19.0	2.28	39.6
Diiodomethane	50.8	50.8	0	0	0

The contact angles *Θ* were measured with the use of the method suggested by Zisman [23], i.e. by means of an optical goniometer (*Cobrabid Optica*–Warsaw) with a digital camera installed in the axial extension of its lens. Those measurements were presented with more details in [19].

## Results and discussion

### Additive SFE model versus polyurethane cationomers structure

The additive effects of surface free energy components (from the van Oss-Good model) and our earlier research [19] made us select the additive model as a tool to describe the influence of the polyurethane chemical structure on the SFE value of the polymer film obtained from that polyurethane. The additive model makes use of a sum of constituent arguments to determine the variable value. That makes it possible to express the parameter *γ*_*S*_ as a sum of independent components (representing the reactants used) and a constant term which is independent on those components. The structure of the model required the following steps to be taken:

1. Categories of input variables were defined which were connected with the types of parent substances and which affected the value of *γ*_*S*_. Each category was formed by a set of reactants employed in the syntheses: diisocyanates, polyols and tertiary amines (with TFBD). HCOOH as quaternisation reagents was used in each synthesis.
2. Reactants in each reactant set were arranged in random order. For example, it can be written down as follows:
  9 $$ \begin{array}{c}\hfill I=\left\{\mathrm{MDI},\mathrm{IPDI}\right\}\hfill \\ {}\hfill J=\left\{\mathrm{POG}600,\mathrm{PPG}450\right\}\hfill \\ {}\hfill K=\left\{\mathrm{NMDA},\mathrm{NBDA},\mathrm{NMDA}+15\%\;\mathrm{TFBD},\dots .\right\}\hfill \end{array} $$
  Where *I* bears information about diisocyjanates, *J* about polyols and *K* tertiary amines with 0-15 % TFBD, respectively.
3. Assuming that each reactant contributes to the value of parameter *γ*_*S*_, further sets *A*, *B* and *C* may be created. They will be composed of elements—contributions of raw materials used in the cationomer synthesis. Hence, the mathematical model which is searched for will have the form of a composite function as below:
  10 $$ {\gamma}_S=A\left[i\right]+B\left[j\right]+C\left[k\right]+\mathrm{const} $$

Where *A*, *B* and *C* are discrete functions which join position of selected reactant within a reactant set: *I*, *J*, *K* with adequate numerical values which represent contributions of those reactants to the value *γ*_*S*_. Successive numbers *i*, *j*, *k* are the pointers of specific reactants used in the synthesis (Table 3), while const parameter represent contribution to the *γ*_*S*_ independent of reactants.

**Table 3:** Polyurethane raw materials denotation

Category of the independent variable	Type of substrate	Substrate in each category	Value of the pointer assigned to the substrate in each category (independent variable)
*I*	Diisocyanate	MDI	1
		IPDI	2
*J*	Polyol	POG 600	1
		PPG 450	2
*K*	Tertiary amines	NBDA + 15%TFBD	1
		NBDA + 10%TFBD	2
		NMDA + 15%TFBD	3
		NBDA + 5%TFBD	4
		NBDA	5
		NMDA + 10%TFBD	6
		NMDA	7
		NMDA + 5%TFBD	8

The numerical estimation of model parameters (10) consists in adjusting the values of all variables *A*[*i*], *B*[*j*], *C*[*k*], and of the parameter const in such a way to minimise deviations between the calculated and experimental values.

The initial point for the search of suitable parameters was selected at random, i.e. from a randomly selected set of initial parameter values A[1, 2, .., *x*], B[1, 2, .., *y*], C[1, 2, .., *z*] and const. Meanwhile, an individual estimation was carried out as follows: after defining a set of initial parameters, a set of values *γ*_*S*_^estim^ was calculated based on Eq. (10), wherein each value had its experimental equivalent in the form of *γ*_*S*_^exp^. Each experimental value of *γ*_*S*_ was compared with the calculated value, which yielded a set of absolute deviations *Δγ* = |*γ*_*S*_^exp^ − *γ*_*S*_^estim^|. It was most convenient to minimise the values of relative deviations, i.e. those which consider also the values of estimated parameters as expressed for example in percent. Therefore, absolute deviations were recalculated to give relative deviations from the following equation:

11 $$ \varDelta {\gamma}_{\%}=\frac{\varDelta \gamma}{\gamma_s^{\exp }}\cdot 100\% $$

Generally, there can be various definitions for the one parameter which yields information of minimum of deviations between the estimated and experimental parameter values within the whole data set. It can be defined, for example, as a minimum in the arithmetical average of a set of deviations, as a minimum of the median or as a minimum of a set of the maximum values of deviation within the set of all deviations. Minimisation of the median value was adopted as the objective function for the needs of this study; therefore, the reasons for that were given in [19].

When the value of deviations set obtained for whole experimental data was calculated as a median, one of the parameters *A*[*i*], *B*[*j*] or *C*[*k*] was selected at random and its value was repeatedly changed (slightly up or down) until the median for the whole set decreased. When the median remained unchanged in successive steps, the arithmetic average values were used instead and the estimation procedure was carried on. After minimisation of the chosen parameter, another parameter (*A*[*i*], *B*[*j*] or *C*[*k*]) was selected at random and procedure was repeated. The sampling and estimation of individual parameters were repeated until there were no further changes noted in the values of any parameters. Finally, the value of the constant parameter (const) was subjected to estimation. Then, the single estimation procedure was completed.

As it was impossible to find the global minimum of the objective function in that procedure, the estimation procedure was repeated many times beginning from any starting point. Sets of estimated parameters obtained in single estimation were stored for reference. The calculations were repeated a lot of times up to 50,000 repetitions. When an estimation cycle was completed, the values of reference deviations for individual estimations were compared to each other. Finally, a set of best matched parameters were selected.

The time of calculation required for a single estimation was primarily dependent on the number of experimental data and the number of parameters to be estimated and obviously on numeric power of computer system. A single estimation lasts from several to 20 min when a typical PC with *i7* processor was employed. A typical data estimation procedure in this study covered 1,000 repetitions, and it took a few days to complete.

When someone has a multiple data set of reacting substances with different *γ*_*S*_^exp^, it is possible to reject the outliers. In case when only single set of the reactants was available, this method enables to detect probable incidental error and helps making decision of rejecting it. The following criterion was adopted for the measurement points, which an estimated value could not deviate from an experimental one by more than 15 %.

In Table 3, designations for the assumed categories of independent variables were presented as well as parent substances belonging to those categories which had been used in the cationomer syntheses. The parent substances were initially assigned arbitrary designations of independent variables (ordinal numbers). Those assignments had to be rearranged more orderly after successive estimations to make the presented model diagrams more clear and legible. The aim was inter alia to avoid “overlapping” of presented curves. These made a basis for 3D diagrams which illustrated the changes in SFE of polyurethane cationomer films versus their chemical compositions. The estimated values of discrete functions A, B and C are presented in Table 4. Table 5, on the other hand, provides the SFE values obtained at the final stage of our numerical calculations of *γ*_S_^estim^ against the experimental values *γ*_S_^exp^. The arithmetic mean of relative deviations between experimental and estimation parameters for whole set of parameters is 3.80 % and median is 2.79 %. Highest individual deviation (13.86 %) was noted for sample no. 6a.

**Table 4:** Parameters for additive model (10) for SFE versus polyurethane cationomers structure calculated in numerical estimation

The value of the independent variable in suitable category	A	B	C
1	5.87	3.72	3.33
2	13.34	9.09	5.14
3			5.40
4			10.20
5			12.64
6			13.70
7			14.90
8			17.34
Const			14.24

**Table 5:** SFE values for cationomer polyurethane films obtained from numerical calculations

Sample no. by Table 1	The value of the independent variable in the category by Table 3			*γ*_S_^exp.^, 0.001 J/m^2^	*γ*_S_^e stim.^, 0.001 J/m^2^	Relative error, %
	Diisocy-anate	Polyol	Tertiary amine or tertiary amine with TFBD			
1	1	1	8	40.30	41.17	2.16
1a	1	1	8	42.91	41.17	4.06
2	1	1	6	38.36	37.53	2.16
2a	1	1	6	36.51	37.53	2.79
3	1	1	3	29.23	29.23	0.00
4	1	1	5	36.47	36.47	0.00
5	1	1	4	34.03	34.03	0.00
6	1	1	2	28.97	28.97	0.00
6a	1	1	2	33.40	28.97	13.26
7	1	1	1	27.16	27.16	0.00
8	1	2	7	42.89	44.10	2.82
8a	1	2	7	44.10	44.10	0.00
9	1	2	5	45.92	41.84	8.89
10	2	1	7	51.12	46.20	9.62
11	2	1	8	48.64	48.64	0.00
12	2	1	6	45.00	45.00	0.00
13	2	1	3	42.07	36.70	12.76
14	2	1	1	37.44	34.63	7.51
15	2	1	5	41.63	43.94	5.55
15a	2	1	5	46.20	43.94	4.89
16	2	1	4	40.16	41.50	3.34
17	2	1	2	36.44	36.44	0.00
18	2	2	7	47.95	51.57	7.55

Mean error = 3.80 %, Median 2.79 %, Max. error = 13.26 %

The calculated parameter values (as specified in Tables 3 and 4) were used in the developed model (10) and the results were analysed. The most important for our consideration is that the results of model calculations are presented in Table 6. For convenience, the values of estimated SFE (*γ*_*S*_^estim^) have been formatted in the last columns in such a way to simplify tracing the changes of SFE following with changing of amine substrate for same combination of other substrates (diisocyanate and polyol). For example, the SFE values of the product of reaction MDI and POG 600 with different amine reagents are adjusted to the left, where product of IPDI, PPG 450 and amines are adjusted to the right.

**Table 6:** Summary of estimated SFE for cationomer coats synthesised from diisocyanates, polyols and tertiary amines with addition of 0–15 % TFBD

Substrates			*γ*_*S*_^estim^			
Tertiary amine (k)	Diisocyanate (i)	Polyol (j)				
NBDA + 15 %TFBD (1)	MDI (1)	POG600 (1)	27.16			
		PPG450 (2)		32.53		
	IPDI (2)	POG600 (1)			34.63	
		PPG450 (2)				40.00
NBDA + 10 %TFBD (2)	MDI (1)	POG600 (1)	28.97			
		PPG450 (2)		34.34		
	IPDI (2)	POG600 (1)			36.44	
		PPG450 (2)				41.81
NMDA + 15 %TFBD (3)	MDI (1)	POG600 (1)	29.23			
		PPG450 (2)		34.60		
	IPDI (2)	POG600 (1)			36.70	
		PPG450 (2)				42.07
NBDA + 5 %TFBD (4)	MDI (1)	POG600 (1)	34.03			
		PPG450 (2)		39.40		
	IPDI (2)	POG600 (1)			41.50	
		PPG450 (2)				46.87
NBDA (5)	MDI (1)	POG600 (1)	36.47			
		PPG450 (2)		41.84		
	IPDI (2)	POG600 (1)			43.94	
		PPG450 (2)				49.31
NMDA + 10 %TFBD (9)	MDI (1)	POG600 (1)	37.53			
		PPG450 (2)		42.90		
	IPDI (2)	POG600 (1)			45.00	
		PPG450 (2)				50.37
NMDA (8)	MDI (1)	POG600 (1)	38.73			
		PPG450 (2)		44.10		
	IPDI (2)	POG600 (1)			46.20	
		PPG450 (2)				51.57
NMDA + 5 %TFBD (8)	MDI (1)	POG600 (1)	41.17			
		PPG450 (2)		46.54		
	IPDI (2)	POG600 (1)			48.64	
		PPG450 (2)				54.01

**Fig. 1:**
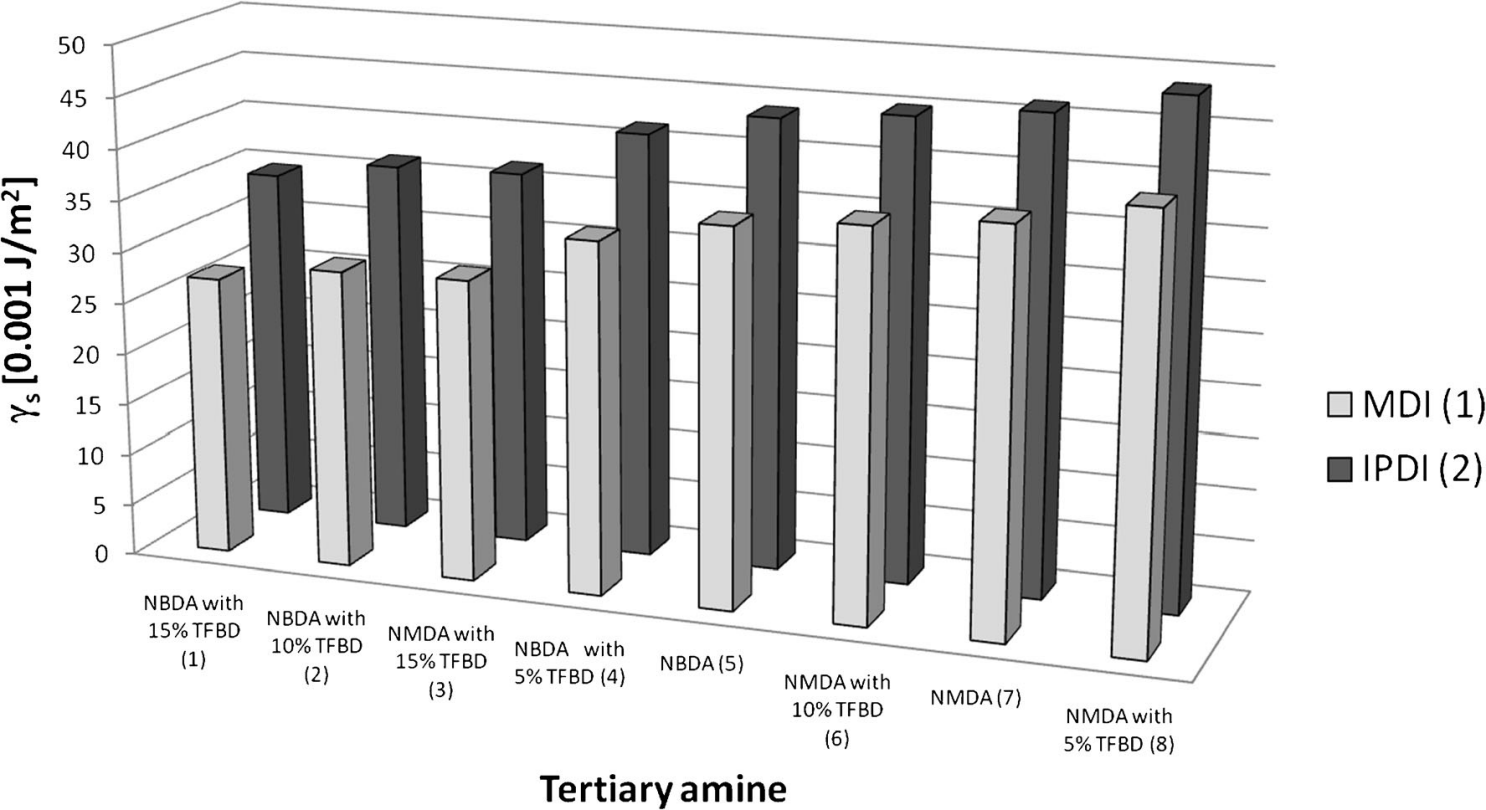
SFE profiles for PU cationomer coats synthesised from POG 600 (*j* = 1) versus types of diisocyanates (*i*) and tertiary amines (*k*)

The conclusion revealed that the value of the parameter sought for (*γ*_*S*_) might vary within ∼25–50 mJ/m^2^ for cationomer films with the structures resulting from the reactants used and their amounts assumed for the synthesis. That range is quite extensive and it covers polymer films which are apolar (hydrophobic) in practice, with γ_S_ < 30 mJ/m^2^, of medium polarity, with 30 < γ_S_ < 40 mJ/m^2^, and definitely polar (hydrophilic) γ_S_ > 40 mJ/m^2^. When the charts presented in Fig. 1 obtained from Eq. (10) are analysed, one finds out that polarity of a polyurethane cationomer film is governed by the structural features which results from all the parent substances used, but the extents of those impacts are different. From the data analysis presented in Table 6 clearly shows that more hydrophobic coatings were obtained from cationomers synthesised from MDI than from IPDI diisocyanate. However, from the two analysed polyols to obtain a more hydrophobic coating, use of POG 600 than the PPG 450 polyol is more advantageous. Identical influence of these diisocyanates was observed for films obtained from linear polyurethanes [19]. However, the biggest effect on reducing the SFE and thus to maintain the strongly hydrophobic coatings are exerting built in to chains *N*-butylammonium cations with HCOO^–^ as counterion without the account to the kind of applied diisocyanate or polyol what is well shown in Table 6. One can see clearly that the hydrophobicity of coatings is enhanced by presence of TFBD in the range of 10–15 wt% in relation to the NBDA, which gives the share of fluoride above 2 % by weight in the material (sample nos. 7 and 14). Obtained in the course of numerical calculations (Table 4) greater value of the parameter C for *k* = 8 (NMDA with 5 % TFBD) than would be expected compared to the value of this parameter for *k* = 7 (NMDA) in our opinion is not justified and may be the result of estimation of experimental data loaded error, but also can attest to the fact that only about 10 % of the TFBD content clearly affects the lower SFE coatings obtained from cationomers based on NMDA amine.

In order to supplement that information and having in mind the considerations as above, we found it advisable to show additionally how specific types of interactions affect the chemical nature of the obtained polymer films. Thus, we provided the calculation results for surface free energy components as obtained from the van Oss-Good model in our earlier research for a few coats with known and clearly different values of *γ*_*S*_^exp^ (for which the errors in *γ*_*S*_^estim^ were not high). As results from the data in Table 7, the highest contribution to the value of *γ*_*S*_ comes from the component *γ*_*S*_^*LW*^—surface energy connected with long-range interactions (dispersion, polar and induction). That group of interactions covers also those of hydrogen bonds interaction. Clearly, one can see that use of fluorinated reactants (sample nos. 3 and 6) reduces the extent of polar interactions, and it yields the films which are definitely hydrophobic and the Lewis-type interactions *γ*_*S*_^*AB*^ in these samples are very low. In the other studied cationomers, contribution of the Lewis-type interactions is generally not too low (below 10 %) and it is probably the result of the presence of ionic groups in the polymer chains.

**Table 7:** Surface free energy components of the van Oss-Good model for selected samples

Sample no. by Table 1	*γ*_S_^exp.^	*γ*_*S*_^*LW*^	*γ*_*S*_^*AB*^
	0.001 J/m^2^	0.001 J/m^2^	0.001 J/m^2^
1	40.30	37.43	2.87
2a	36.51	33.35	3.16
3	29.23	29.11	0.12
4	36.47	34.92	1.55
6	28.97	28.48	0.36
16	40.16	39.02	1.14
18	47.95	47.90	0.05

## Conclusions

Structural modifications of polyurethane cationomers with the use of various diisocyanates, polyols and tertiary amines with quaternisation reagents (alkyl bromides or formic acid) make it possible to produce polymer coats with the SFE values above 30 mJ/m^2^. When additional fluoride atoms are introduced, e.g. in the form of specially synthesised fluoric polyols like for example POG 2000 fluoric or when TFBD together with tertiary amines are introduced, the SFE value can be reduced down to below 20 mJ/m^2^. The chemical nature of a coat is controlled to a considerable extent also by the type of ion groups derived from tertiary amines by quaternisation reagents and by counter ions, in this case HCOO^–^ anions, which have been built-in into polymer chains. The highest contribution to SFE comes from the component *γ*_*S*_^*LW*^, i.e. from long-range interactions, but no Lewis interactions *γ*_*S*_^*AB*^ may be neglected for cationomers since their contributions to the total value of *γ*_*S*_ may be as high as about 20 % in some cases.

Summing up the additive model developed within this study seems to be able to reliably predict the SFE values of polymer coats according to the cationomer chain structures which are controlled by the types of initial reacting substances. The method which has been worked out is general enough to be employed in analysing polarity of other polymer systems which are used to form films and coats. However, one should always remember about the limitations for estimation methods which make use of experimental data. Certainly, the quality and volume of the available experimental material is always a key to providing adequate quality of calculations. Hence, if we had a higher number of reliable experimental data points, we could go on with the estimation procedure to establish more precise values of the studied parameters and possibly to expand the scope of our research and to involve new parent substances. That additional research could change somewhat the sequence of parameters within each category *i*, *j*, *k* sorted out according to their values. That is more and more probable for lower and lower differences in the values of successive parameters as listed in Table 4. However, general conclusions which result from this study are not expected to be changed by any modification in the sequence of parameters.
